# The Distinction of Clinicopathological Characteristics, Treatment Strategy and Outcome in Colorectal Cancer Patients With Synchronous vs. Metachronous Bone Metastasis

**DOI:** 10.3389/fonc.2020.00974

**Published:** 2020-06-19

**Authors:** Chen-xi Ma, Xu Guan, Ran Wei, Song Wang, Ji-chuan Quan, Zhi-xun Zhao, Hai-peng Chen, Zheng Liu, Zheng Jiang, Xi-shan Wang

**Affiliations:** ^1^Department of Colorectal Surgery, National Cancer Center/National Clinical Research Center for Cancer/Cancer Hospital, Chinese Academy of Medical Sciences and Peking Union Medical College, Bejing, China; ^2^Department of Colorectal Surgery, The Second Affiliated Hospital of Harbin Medical University, Harbin, China

**Keywords:** colorectal cancer, bone metastasis, synchronous, metachronous, cancer specific survival, prognostic factor

## Abstract

**Background:** The impact of the timing of bone metastasis (BM) diagnosis on colorectal cancer (CRC) patients is unclear. Our study aimed to explore the differences in clinicopathological characteristics, treatments and prognosis between synchronous BM (SBM) and metachronous BM (MBM) from CRC.

**Methods:** We retrospectively investigated clinical data of CRC patients with SBM or MBM from 2008 to 2017 at Chinese National Cancer Center. Cancer specific survival (CSS) after BM diagnosis was estimated using the Kaplan-Meier method. The multivariable COX regression model identified the prognostic factors of CSS.

**Results:** Finally, 63 CRC patients with SBM and 138 CRC patients with MBM were identified. Compared to SBM from CRC, MBM significantly was more involving multiple bone lesions (63.0 vs. 7.9%; *p* < 0.001), and more frequently originated from rectal cancer (60.9 vs. 41.3%; *p* = 0.033). The therapeutic strategies in SBM and MBM group were contrasted including systemic treatment, bisphosphonates, radiotherapy and metastasectomy for BM. 85.5% of patients in MBM group and 25.4% of patients in SBM group underwent primary tumor resection at initial diagnosis (*p* < 0.001). The median CSS was 11 months in both SBM and MBM group (*p* = 0.556), yet MBM patients developed from CRC in early AJCC stage presented obviously longer survival than those from advanced stage. Furthermore, patients could have improved CSS from primary tumor resection while there might be no survival benefit from targeted therapy in both SBM and MBM groups. Bisphosphonates was associated with a better CSS for patients with SBM, while radiotherapy for BM was related to a better CSS for patients with MBM.

**Conclusion:** The CRC patients in SBM and MBM group represented different clinicopathological characteristics and treatment modalities, which affected the prognosis in different ways. Distinct consideration for CRC patients with SBM and MBM in clinical decision making is required.

## Introduction

Colorectal cancer (CRC) with distant metastasis is one of the main causes of death. About 20% of CRC patients are diagnosed with distant metastasis at initial diagnosis and 50–60% will eventually have metastases (1, 2). The CRC commonly metastasizes to liver, followed by lung, yet seldom to bone (3). Population-based studies have reported the incidence of BM is 3.0–10.4% in CRC patients (4–6), but previous autopsy findings have suggested incidence of up to 23.7% (7). The prognosis after BM detection is generally poor due to the advanced stage and the difficulty in treatment, with 5-year survival rate < 5% (8). Median overall survival of CRC patients after BM diagnosis ranges from 5 to 22 months according to most researches (4, 9), with diverse factors affecting their prognosis such as some clinicopathological characteristics and provision of treatment. However, there is a lack of standard treatment guideline for BM from CRC at present. The possible therapies for BM include systemic therapy, local therapy and supportive treatment, with purpose to prevent skeletal-related events (SREs) like sever bone pain, hypercalcemia, spinal cord compression and pathological fracture and improve the survival of patients.

Synchronous BM (SBM) in CRC patients is relatively rare while most BMs occur metachronously after a length of follow-up time or during palliative treatment for other metastases. Generally, the patients with metachronous BM (MBM) have received systematic clinical intervention before the osseous lesion development, whereas those with SBM are mostly naive. Therefore, SBM and MBM from CRC may represent distinct clinicopathological characteristics, therapeutic sensitivity and outcomes, which require different treatment strategies. Many reports are controversial on the outcomes of synchronous and metachronous metastases from CRC, and most of which agree about the more aggressive clinical and pathological features of synchronous metastases (10–14). However, few studies in specifically exploring the differences between SBM and MBM from CRC have been reported.

Thus, the aims of our study were to (1) compare the clinicopathological characteristics of SBM and MBM from CRC; (2) compare the treatment modalities for SBM and MBM from CRC; (3) explore outcomes and prognostic factors of CRC patients with SBM and MBM, especially the impact of various treatment modalities on their prognosis, which would be helpful in modifying clinical management.

## Materials and Methods

### Data Resources and Study Population

CRC patients who were diagnosed with BM between January 2008 and December 2017 at Chinese National Cancer Center, were retrospectively identified. The primary CRC lesion was confirmed by histopathological examination. The American Joint Committee on Cancer (AJCC) TNM stage and BM were identified by histopathological or imaging examinations such as standard X-rays, whole-body bone scans, computed tomography (CT), magnetic resonance imaging (MRI) and positron emission tomography-computed tomography (PET-CT). SBM refers to BM found within 3 months after the diagnosis of CRC, while MBM refers to BM found more than 3 months after the diagnosis of CRC (15, 16). For the number of BM, two adjacent vertebral metastases were classified into the solitary bone involvement, while non-consecutive metastases or more than 2 consecutive vertebral metastases were classified as multiple bone involvement. The time of follow-up was calculated from the BM diagnosis to death or January 2020. The cancer specific survival (CSS) was defined as the time from the BM diagnosis until cancer-associated death or the end of follow up. This study was approved by the Ethics Committee of Cancer Hospital, Chinese Academy of Medical Sciences.

### Prognostic Factors

Clinicopathological data and treatment methods were collected from medical records or via telephone follow-ups. Common variables were analyzed including age, gender, basic disease, primary tumor location, pathological type of tumor, tumor grade, carcinoembryonic antigen (CEA) levels at BM diagnosis, carbohydrate antigen199 (CA199) levels at BM diagnosis, alkaline phosphatase (ALP) levels at BM diagnosis, bone involvement, Karnofsky performance scores (KPS) at BM diagnosis, extra-osseous metastases, primary tumor resection, systemic treatment for BM, bisphosphonates for BM, radiotherapy for BM and operation for BM. Besides, AJCC TNM stage at initial diagnosis and time until BM were additionally evaluated for MBM. The basic disease was defined as other long-term or chronic coexisting diseases the BM patients from CRC suffered from, which affected basic metabolism or immune function of patients, mainly including hypertension, diabetes, heart disease, hepatitis, tuberculosis, autoimmune diseases, etc.

### Statistical Analysis

The comparison of clinicopathological characteristics and treatments between patients with SBM and MBM was done using χ2-test or Fisher's exact test where appropriate. The CSS was assessed with Kaplan-Meier method, with the log-rank tests used to compare subgroups. In order to reduce the selection bias, variables with *p* < 0.10 by univariate Kaplan-Meier analysis were selected first, then a forward stepwise selection was performed using the selected variables in multivariable COX regression analysis. The independent prognostic factor was defined as the variable with *p* < 0.05 by COX regression. Hazard ratio (HR) and corresponding 95% confidence interval (CI) were also calculated by multivariable COX analysis. All statistical analyses were performed with SPSS version 25.0 for Mac. It is considered as statistically significant when *p* < 0.05.

## Results

**Figure 1:**
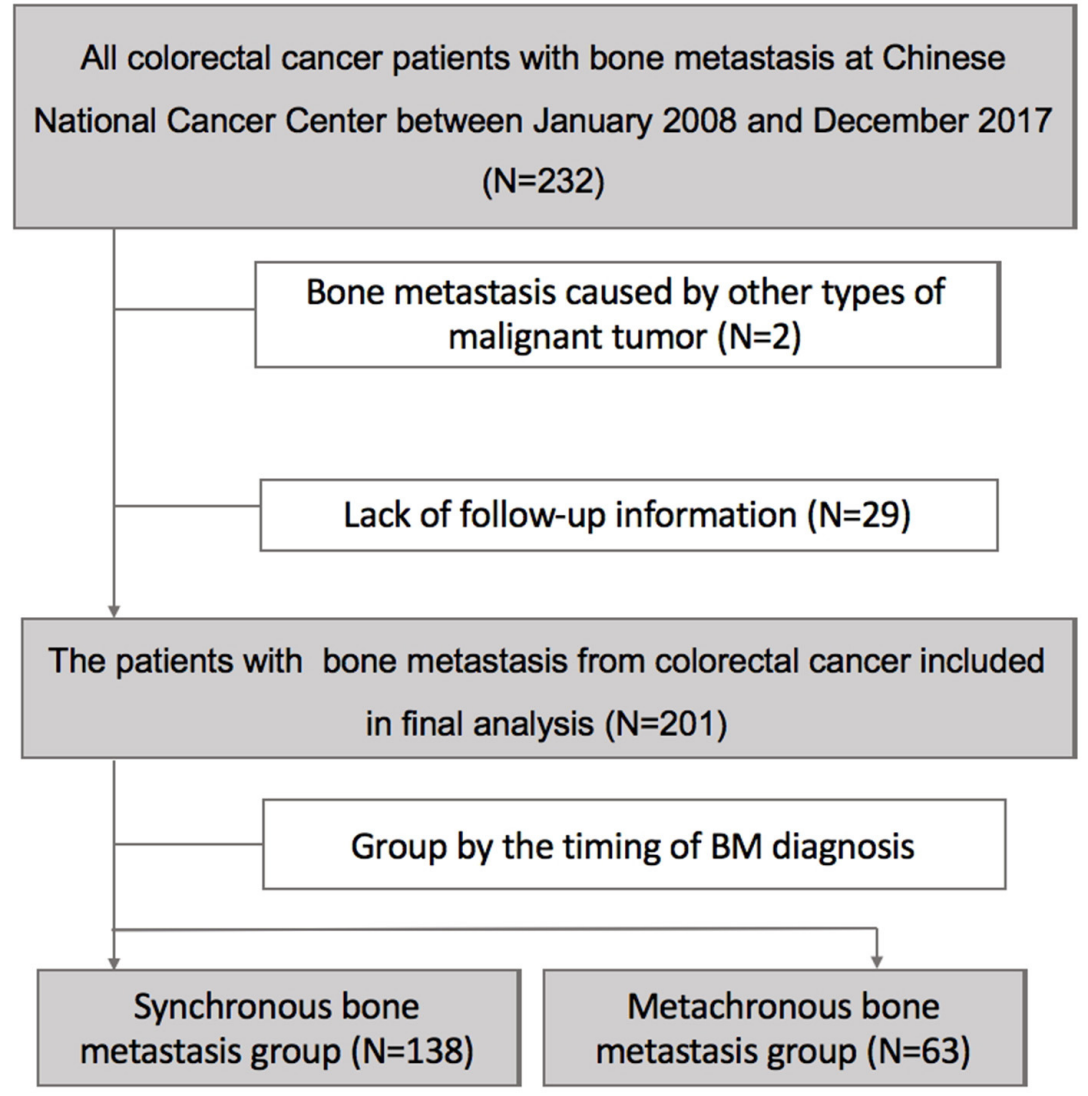
The analytical cohort and exclusion criteria.

Finally, in total of 201 patients diagnosed with BM from CRC entered in our final analysis after excluding 31 cases who were not eligible (Figure 1). 31.3% of patients (63/201) were identified with SBM at initial diagnosis while additional 68.7% of patients (138/201) developed MBM after diagnosis of CRC.

### Patients Characteristics

Table 1 represented the clinicopathological characteristics of CRC patients with SBM and MBM. The median age of patients was 58 years (range 33–84) in SBM group and 59 years (range 19–79) in MBM group, respectively. CRC patients with SBM and MBM were similar with respect to their age at BM diagnosis (*p* = 0.974), gender (*p* = 0.459) and basic disease (*p* = 0.628). The rectal cancer was more common in MBM group (60.9%) than SBM group (41.3%), with statistical significance (*p* = 0.033). Patients with MBM were diagnosed more often with lower tumor grade (63.8 vs. 46.0%; *p* = 0.048) compared to those with SBM. Performances in CAE levels (*p* = 0.511), CA199 levels (*p* = 0.619), ALP levels (*p* = 0.827) and KPS (*p* = 0.631) at BM diagnosis between two groups were, respectively similar.

**Table 1:** The comparison of clinicopathological characteristics in CRC patients with SBM and MBM.

**Variable**	**Synchronous**		**Metachronous**		***p-*value**
	***N =* 63**	**%**	***N =* 138**	**%**	
**Age at BM diagnosis, years**					0.974
< 60	35	55.6	77	55.8	
≥ 60	28	44.4	61	44.2	
**Gender**					0.459
Female	23	36.5	58	42.0	
Male	40	63.5	80	58.0	
**Basic disease**					0.628
No	37	58.7	86	62.3	
Yes	26	41.3	52	37.7	
**Primary tumor location**					0.033
Rectum	26	41.3	84	60.9	
Left hemicolon	17	27.0	23	16.6	
Right hemicolon	20	31.7	31	22.5	
**Pathological type of tumor**					0.054^*^
Adenocarcinoma	56	88.9	128	92.8	
Signet-ring cell carcinoma	4	6.3	10	7.2	
Others	3	4.8	0	0.0	
**Tumor grade**					0.048^*^
Grade I, II	29	46.0	88	63.8	
Grade III, IV	19	30.2	31	22.4	
UK	15	23.8	19	13.8	
**AJCC TNM stage at initial diagnosis**					<0.001^*^
I	0	0.0	6	4.3	
II	0	0.0	14	10.2	
III	0	0.0	73	52.9	
IV	63	100.0	41	29.7	
UK	0	0.0	4	2.9	
**CEA levels at BM diagnosis**					0.511
Negative	16	25.4	28	20.3	
Positive	41	65.1	90	65.2	
UK	6	9.5	20	14.5	
**CA199 levels at BM diagnosis**					0.619
Negative	27	42.9	50	36.2	
Positive	29	46.0	68	49.3	
UK	7	11.1	20	14.5	
**ALP levels at BM diagnosis**					0.827
Negative	47	74.6	102	73.9	
Positive	14	22.2	29	21.0	
UK	2	3.2	7	5.1	
**Bone involvement**					<0.001
Solitary	58	92.1	51	37.0	
Multiple	5	7.9	87	63.0	
**KPS at BM diagnosis**					0.631
≥ 80	49	77.8	103	74.6	
< 80	14	22.2	35	25.4	
**Extra-osseous metastases**					0.959
No	7	11.1	15	10.9	
Yes	56	88.9	123	89.1	
**Time until BM**					-
3 months−1 year	-	-	45	32.6	
1–3 years	-	-	69	50.0	
>3 years	-	-	24	17.4	

No marks indicated the p-value was calculated by Chi-square test and an asterisk (^*^) indicated the p-value was calculated by Fisher's test. CRC, colorectal cancer; SBM, synchronous bone metastasis; MBM, metachronous bone metastasis; N, number; UK, unknown; CEA, carcinoembryonic antigen; CA199, carbohydrate antigen199; ALP, alkaline phosphatase.

### Patterns of BM and Extra-Osseous Metastasis

Patients with MBM (63.0%) were significantly more involving multiple bone lesions compared to those with SBM (7.9%; *p* < 0.001). Spine (65.1 vs. 73.2%) was the leading site of BM in SBM and MBM group, followed by pelvis (57.1 vs. 62.0%), long bones (34.9 vs. 22.6%) and ribs (30.2 vs. 21.9%).

There were 88.9% of patients (56/63) in SBM group and 89.1% of patients (123/138) in MBM group having extra-osseous metastases, respectively, with no significant difference (*p* = 0.959). The common extra-osseous sites were liver (61.9%), distant lymph nodes (54.0%) and lung (39.7%) in SBM patients. While lung (57.7%) was the most common extra-osseous metastatic site in MBM patients, followed by liver (45.7%) and lymph nodes (40.9%).

### Treatments

There were 85.5% of MBM patients (118/138) receiving primary tumor resection, which had been all performed at initial diagnosis. The proportions of primary tumor resection in MBM patients with AJCC stage I, II, III and IV were 100.0% (6/6), 100.0% (14/14), 97.3% (71/73), and 58.5% (24/41), respectively. Of the four patients with unknown AJCC TNM stage, three received this operation. In SBM group, only 25.4% of patients (16/63) underwent primary tumor resection because of the advanced stage.

All patients received palliative chemotherapy after BM diagnosis. There were 37.9% of cases in SBM group (25/63) and 39.1% of cases in MBM group (54/138; *p* = 0.941) receiving additional targeted therapy, respectively. The proportions of patients who received bisphosphonates treatment (60.3 vs. 55.8%; *p* = 0.548) or radiotherapy (77.8 vs. 68.8%; *p* = 0.192) were, respectively, similar between two groups. Only 2.2% of patients with MBM (3/138) underwent operative treatment for BM due to spinal cord compression while no patient with SBM received metastasectomy for BM (*p* = 0.541). The details were shown in Table 2.

**Table 2:** The comparison of treatment strategies in CRC patients with SBM and MBM.

**Variable**	**Synchronous**		**Metachronous**		***p*-value**
	***N =* 63**	**%**	***N =* 137**	**%**	
**Primary tumor resection**					<0.001
No	47	74.6	20	14.5	
Yes	16	25.4	118	85.5	
**Systemic treatment after BM diagnosis**					0.941
Chemotherapy alone	38	60.3	84	60.9	
Chemotherapy plus targeted therapy	25	39.7	54	39.1	
**Bisphosphonates for BM**					0.548
No	38	60.3	77	55.8	
Yes	25	39.7	61	44.2	
**Radiotherapy for BM**					0.192
No	49	77.8	95	68.8	
Yes	14	22.2	43	31.2	
**Metastasectomy for BM**					0.553^*^
No	63	100.0	135	97.8	
Yes	0	0.0	3	2.2	

No marks indicated the p-value was calculated by Chi-square test and an asterisk (^*^) indicated the p-value was calculated by Fisher's test. CRC, colorectal cancer; SBM, synchronous bone metastasis; MBM, metachronous bone metastasis; N, number.

### Survival

**Figure 2:**
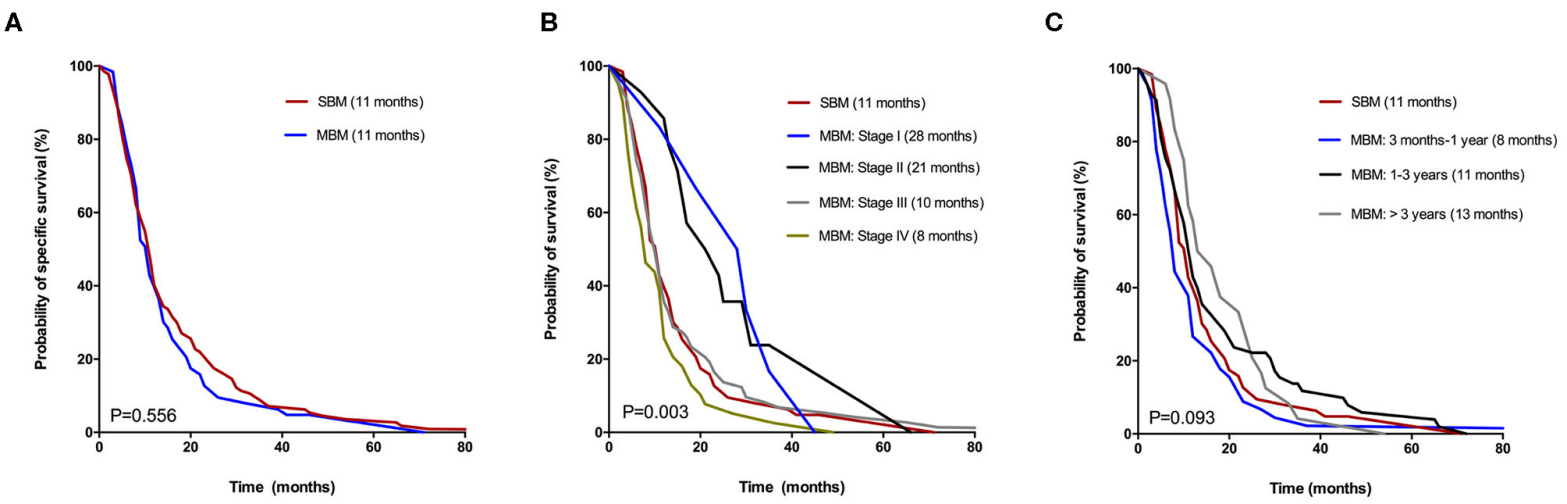
Kaplan-Meier curves for CSS of CRC patients with SBM and MBM according to different situations: **(A)** Overall CSS. **(B)** AJCC TNM stage at initial diagnosis. **(C)** Time until diagnosis of BM.

In total of 195 CRC patients (97.0%) died because of cancer during a median follow-up time of 11 (range 1–198) months, with 61 cases in SBM group and 134 cases in MBM group. And only one patient with MBM died due to other disease. Median CSS was both 11 months for patients with SBM and MBM. The median interval time from CRC diagnosis to MBM was 18.5 months. Figure 2 displayed the Kaplan-Meier curves of SBM and MBM group according to different situations. The overall CSS of patients with SBM and MBM was similar, with no significant difference (*p* = 0.556; Figure 2A). The median CSS in MBM patients with AJCC stage I, II, III and IV at initial CRC diagnosis was 28, 21, 10, and 8 months, respectively, which also showed large differences compared to SBM patients **(***p* = 0.003; Figure 2B). In addition, patients diagnosed with MBM >3 years after CRC diagnosis had a similar CSS with SBM patients (*p* = 0.093; Figure 2C).

**Figure 3:**
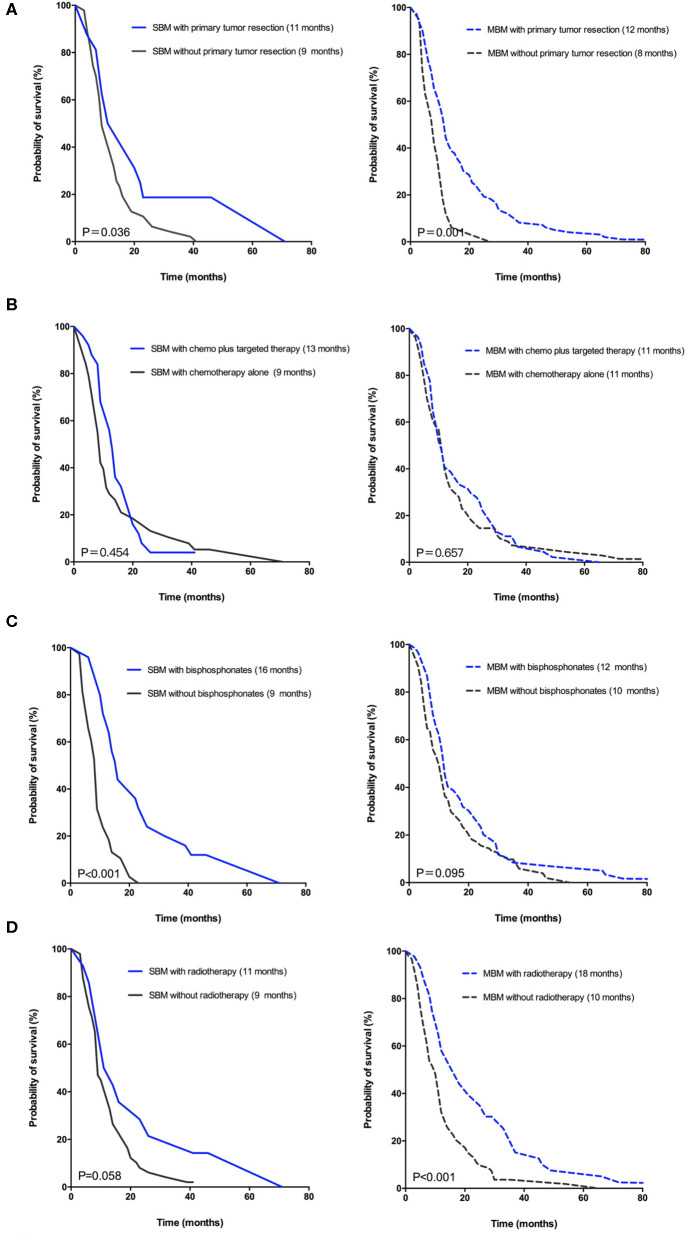
Kaplan-Meier curves for CSS of CRC patients in synchronous group (?) versus metachronous group (—) according to various treatments: **(A)** Primary tumor resection. **(B)** Systemic treatment after BM diagnosis. **(C)** Bisphosphonates for BM. **(D)** Radiotherapy for BM.

To elucidate the outcomes with various treatments in two groups, the Kaplan-Meier curves for SBM and MBM patients were, respectively, represented in Figure 3. Patients who had underwent primary tumor resection at initial diagnosis in SBM or MBM group (Figure 3A) both had a better survival. The CSS was no significantly different between patients with and without targeted therapy in both two groups (Figure 3B). Bisphosphonates therapy was related to a better CSS in synchronous group (Figure 3C) while radiotherapy for BM (Figure 3D) was related to a better CSS in MBM group. Because only 3 patients took the osseous metastasectomy, the relationship between operation for BM and CSS was unclear.

### Prognostic Factors

**Figure 4:**
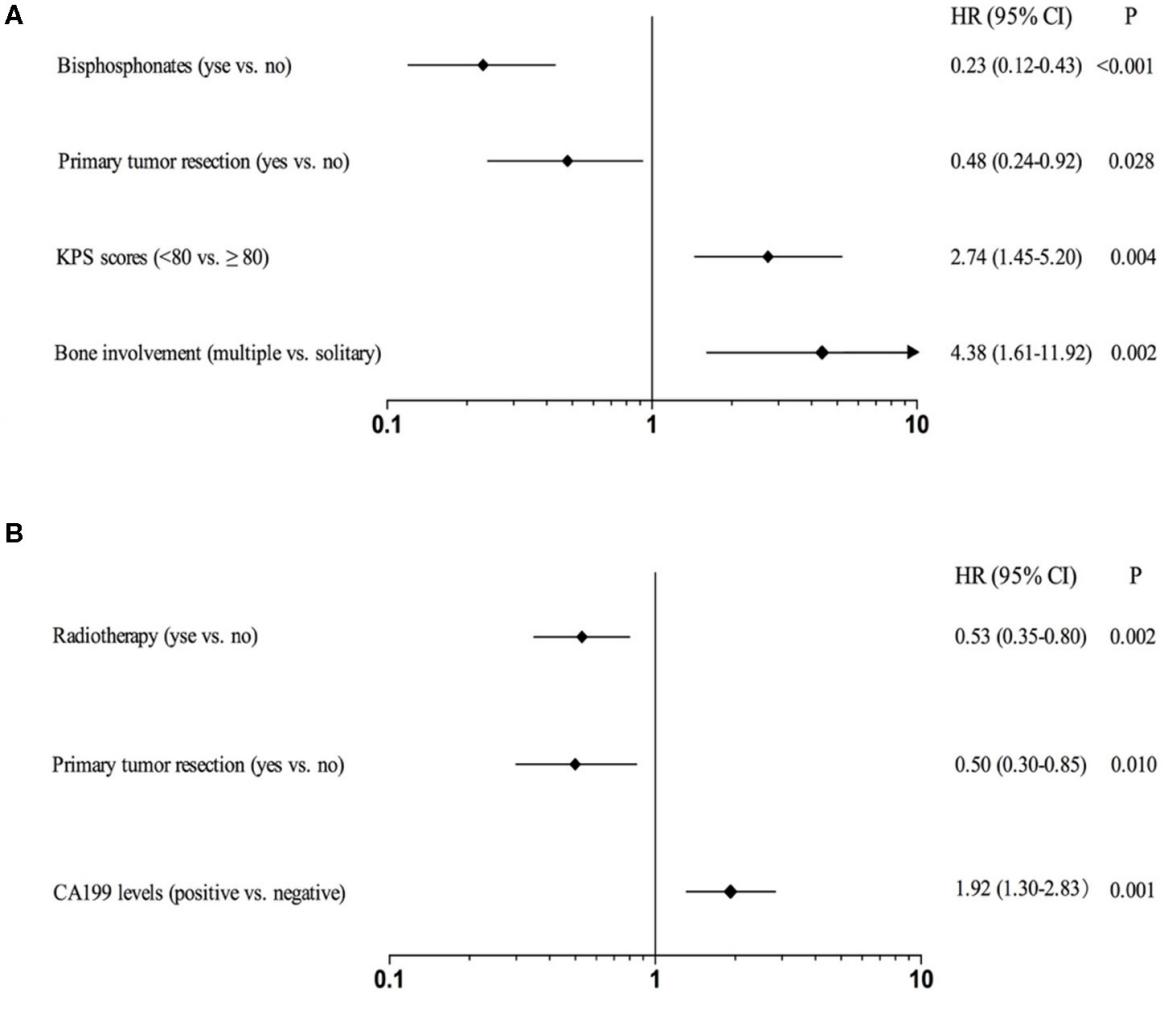
Forest plots for CSS of CRC patients with SBM **(A)** or MBM **(B)** based on multivariable COX proportional hazard model.

Table 3 showed the *p*-values obtained by univariate Kaplan-Meier analysis in SBM and MBM group, respectively. And the variables with *p* < 0.10 were selected to be further analyzed. The independent prognostic factors (*p* < 0.05) were finally identified by multivariable COX regression analysis. We found multiple bone involvement (HR: 4.38; 95%CI: 1.61–11.92; *p* = 0.002), KPS scores <80 (HR: 2.74; 95%CI: 1.45–5.20; *p* = 0.004), primary tumor resection (HR: 0.48; 95%CI: 0.24–0.92; *p* = 0.028) and bisphosphonates (HR: 0.23; 95%CI: 0.12–0.43; *p* < 0.001) were independent prognostic factors for SBM patients (Figure 4A). While positive CA199 levels (HR: 1.92; 95%CI: 1.30–2.83; *p* = 0.001), primary tumor resection (HR: 0.50; 95%CI: 0.30–0.85; *p* = 0.010) and radiotherapy (HR: 0.53; 95%CI: 0.35–0.80; *p* = 0.002) were independent prognostic factors for MBM patients (Figure 4B).

**Table 3:** The univariate Kaplan-Meier analysis in CRC patients with SBM and MBM.

**Variable**	**Synchronous**			**Metachronous**		
	**Median CSS (months)**	**95% CI**	***p*-value**	**Median CSS (months)**	**95% CI**	***p*-value**
**Age at BM diagnosis, years**			0.564			0.521
< 60	9	8.2–9.8		11	9.0–13.0	
≥ 60	11	7.9–14.1		12	10.7–13.4	
**Gender**			0.617			0.705
Female	10	7.7–12.3		11	8.8–13.2	
Male	11	8.3–13.7		11	9.6–12.4	
**Basic disease**			0.494			0.209
No	11	9.2–12.8		12	10.5–13.5	
Yes	9	6.0–12.0		11	9.3–12.7	
**Primary tumor location**			0.990			0.273
Rectum	11	8.0–14.0		12	10.3–13.7	
Left hemicolon	11	6.0–16.0		11	7.9–14.1	
Right hemicolon	9	9.5–12.5		9	6.4–11.6	
**Pathological type of tumor**			0.766			0.516
Adenocarcinoma	11	8.6–13.4		11	9.8–12.2	
Signet-ring cell carcinoma	4	1.1–6.9		8	5.0–11.0	
Others	8	1.6–14.4		NA	NA	
**Tumor grade**			0.494			0.539
Grade I, II	13	9.0–17.0		12	10.7–13.3	
Grade III, IV	9	8.2–9.8		8	5.9–10.1	
UK	9	5.2–12.8		9	5.4–12.6	
**AJCC TNM stage at initial diagnosis**			NA			0.003^*^
I	NA	NA		28	14.8–41.2	
II	NA	NA		21	8.2–33.8	
III	NA	NA		10	8.2–11.8	
IV	11	9.6–12.4		8	4.3–11.7	
UK	NA	NA		11	0.0–29.6	
**CEA levels at BM diagnosis**			0.139			0.008^*^
Negative	11	7.1–14.9		11	8.9–13.1	
Positive	10	8.1–11.9		11	9.2–12.8	
UK	8	0.0–16.4		12	6.2–17.8	
**CA199 levels at BM diagnosis**			0.367			<0.001^*^
Negative	11	7.6–14.4		13	9.0–17.0	
Positive	9	8.0–10.0		8	6.5–9.5	
UK	13	0.2–25.8		12	6.2–17.8	
**ALP levels at BM diagnosis**			0.023^*^			0.024^*^
Negative	11	7.6–14.4		12	10.9–13.1	
Positive	8	5.6–10.4		8	2.7–13.3	
UK	4	NA		10	4.9–15.1	
**Bone involvement**			0.001^*^			0.034^*^
Solitary	11	8.9–13.1		15	10.4–19.6	
Multiple	5	3.9–6.1		10	8.0–12.0	
**KPS at BM diagnosis**			0.001^*^			0.446
≥ 80	11	8.1–13.9		12	10.7–13.3	
<80	5	1.3–8.7		7	1.3–12.7	
**Extra-osseous metastases**			0.603			0.224
No	11	8.7–13.3		12	5.4–18.6	
Yes	10	8.2–11.8		11	9.4–12.6	
**Time until BM**			NA			0.063^*^
3 months−1 year		NA		8	6.7–9.3	
1–3 years		NA		12	10.4–13.6	
>3 years		NA		13	7.0–19.0	
**Primary tumor resection**			0.036^*^			0.001^*^
No	9	7.0–11.0		8	1.2–10.8	
Yes	11	3.2–18.8		12	10.7–13.3	
**Systemic treatment after BM diagnosis**			0.454			0.657
Chemotherapy alone	9	7.8–10.2		11	9.7–12.3	
Chemotherapy plus targeted therapy	13	11.0–15.0		11	8.8–13.2	
**Bisphosphonates for BM**			<0.001^*^			0.095
No	9	8.0–10.0		10	7.4–12.6	
Yes	16	12.8–19.2		12	10.3–13.7	
**Radiotherapy for BM**			0.058^*^			<0.001^*^
No	9	7.6–10.4		10	8.1–11.9	
Yes	11	5.5–16.5		18	11.6–24.4	
**Metastasectomy for BM**			NA			0.541
No	NA	NA		11	9.6–12.4	
Yes	NA	NA		21	5.0–37.0	

An asterisk (^*^) indicated variables with p-value < 0.10, which was selected into multivariable COX regression. CRC, colorectal cancer; SBM, synchronous bone metastasis; MBM, metachronous bone metastasis; CI, confidence interval; NA, not available; CEA, carcinoembryonic antigen; CA199, carbohydrate antigen199; ALP, alkaline phosphatase.

## Discussion

To our knowledge, this study is the first to retrospectively analyze the SBM and MBM together from CRC patients. The MBMs were more common, with incidence nearly twice higher than SBMs. The clinicopathological characteristics differed between two groups. The most striking finding was that in total of 63.0% of patients in MBM group had BMs to multiple sites, far more than those (7.9%) in SBM group. That might be because liver metastasis or lung metastasis from CRC in MBM group would have enough time to spread to skeletal systems by systemic circulation or directly invade chest bones such as sternum, rib and clavicle. We found there were similar therapeutic strategies between two groups, except that more MBM patients received the resection of primary tumor at initial diagnosis.

Colloca et al. (14) identified 425 CRC patients with distant metastases, discovering that the survival after metastasis diagnosis was shorter in synchronous group (18.5 vs. 62.8 months, *p* < 0.001). Majority of reports consider synchronous metastases from CRC to be more aggressive than metachronous despite there is a controversy (10–14). In our study, the prognosis of BM was very poor, yet there was no significant difference in CSS between two groups (Figure 2A). Several reasons might explain the similar outcomes. First, a significant percentage of patients with MBM had been treated with prior chemotherapy before BM diagnosis, while patients with SBM obviously were not and they were more chemo-naive chemo-sensitive (13, 14). Second, the multiple bone involvement was related to worse prognosis, which was more common in MBM group. Another possibility was that BMs was so aggressive that the timing of BM diagnosis had little impact on the outcome. In addition, patients with different time intervals to MBM diagnosis had similar CSS with SBM patients as Figure 2C represented. However, it was difficult to interpret this result because the CSS of MBM patients significantly varied by different AJCC stages.

The clinical outcomes of patients with SBM and MBM appeared to be affected by different clinicopathological characteristics. We found CA199 levels was an independent prognostic factor only for MBM patients. But the CSS of patients with positive ALP levels was shorter in both two groups by univariate analysis, which was consistent with previous studies (13). So, careful surveillance in those indicators for patients with BM from CRC is recommended. Most researches have revealed the relationship between multiple BMs and worse prognosis (17, 18), while minority of studies have demonstrated the prognosis of CRC patients has no association with the number of BMs (19). In our study, multiple bone involvement was related to shorter survival. The median CSS of patients with multiple and solitary bone involvement was 5 and 11 months in SBM group (*p* < 0.001) and 10 and 15 months in MBM group (*p* = 0.034), respectively. Therefore, systematic imaging examination is helpful to assess the outcome of BM.

The association between TNM stage and overall survival of CRC is generally confirmed (20). In our study, MBM patients with stage I at initial diagnosis had best prognosis with median CSS of 28 months, while it dramatically decreased to 8 months for those with stage IV (*p* = 0.003). Thus, strengthening early diagnosis of CRC and active treatment might also prolong the CSS even the BM was developed metachronously.

The prognosis of patients with SBM and MBM was also affected by distinct provision of treatment. As the rare metastatic disease, standard treatment guidelines for CRC patients with BM have not been established. Because all cases were treated with palliative chemotherapy after BM diagnosis in our study, the utility of chemotherapy in each group was unclear.

Bisphosphonates therapy can prevent the occurrence of osteolytic lesions and SREs caused by BM, which has become an effective treatment for bone pain and hypercalcemia (21, 22). Commonly used bisphosphonates such as pamidronate, zoledronic acid and ibandronate can be treated for BM patients in combination with conventional anti-tumor drugs. The difference in CSS of patients with and without bisphosphonates is significant only in SBM group, implying the sensitivity to bisphosphonates for SBM and MBM patients might exist difference.

Local treatments of CRC with BM include radiotherapy and surgery, etc. Previous researches have revealed radiotherapy can reduce bone pain and prevent pathological fracture or spinal cord compression (23–25). According to our study, median CSS of patients with palliative radiotherapy was significantly prolonged only in MBM group (18 vs. 10 months, *p* < 0.001), which was also found to be one of independent prognostic factors for MBM patients. A meta-analysis of 1,026 cases from retrospective studies had suggested an improved survival for stage IV CRC patients with primary tumor resection (26). Another recent research enrolled 3,423 patients, reporting a poor prognosis for the patients with synchronous metastases who did not receive the resection of primary tumor (27). Despite these evidences, primary tumor resection has not been confirmed as a factor related to prolonged outcome of patients with unresectable synchronous metastases (28, 29). Our study showed that patients with primary tumor resection had significantly longer CSS in SBM group (11 vs. 9 months, *p* = 0.036), which was also an independent prognostic factor. This might be attributable to the reduction of tumor-related complications such as systemic inflammation, bleeding, obstruction and perforation. If advanced patients can tolerate the operation, active treatment for CRC is an alternative method for improving the survival of BM (30). Only 3 patients underwent operative treatment of BM due to spinal cord compression in our study and we could not evaluate its' effect on outcome of patients. When non-operative treatment for pathological fracture, spinal instability or other complications caused by BM is invalid, surgical treatment for BM could be considered.

Our study had some limitations. First, this was a retrospective and single-center study, selection bias might occur. Second, the modest samples and non-randomized design limited the generalizability for the conclusions regarding optimal clinical management. Therefore, further prospective researches with randomized design, large sample and more clinical features are warranted.

## Conclusion

Our study compared the clinical data and outcomes of SBM and MBM patients from CRC. Meanwhile, we identified favorable clinicopathological characteristics and treatments in SBM and MBM group, respectively. That could potentially guide physicians to treat patients with distinct clinical intervention and therapeutic strategies.

## Data Availability Statement

The datasets generated for this study are available on request to the corresponding author.

## Ethics Statement

The studies involving human participants were reviewed and approved by Ethics Committee of National Cancer Center/Cancer Hospital, Chinese Academy of Medical Sciences. Written informed consent for participation was not required for this study in accordance with the national legislation and the institutional requirements.

## Author Contributions

XW: writing-review and editing, supervision. XG: conceptualization, investigation, and supervision. CM: conceptualization, investigation, and writing-original draft. RW: software and formal analysis. SW: validation and data curation. JQ: resources. ZZ: validation. HC: investigation and project administration. ZL: data curation and supervision. ZJ: data curation and revision. All authors contributed to the article and approved the submitted version.

## Conflict of Interest

The authors declare that the research was conducted in the absence of any commercial or financial relationships that could be construed as a potential conflict of interest.
